# Molecular basis and therapeutic implications of binary YAP^On^/YAP^Off^ cancer classes

**DOI:** 10.1042/BCJ20253077

**Published:** 2025-05-28

**Authors:** Pinky Sharma, Yale S. Michaels, Joel D. Pearson

**Affiliations:** 1 Department of Pharmacology & Therapeutics, Max Rady College of Medicine, University of Manitoba, Winnipeg, MB, Canada; 2 Paul Albrechtsen Research Institute CancerCare Manitoba, Winnipeg, MB, Canada; 3 Department of Biochemistry & Medical Genetics, Max Rady College of Medicine, University of Manitoba, Winnipeg, MB, Canada; 4 Children’s Hospital Research Institute of Manitoba, Winnipeg, MB, Canada

**Keywords:** cancer stratification, leukemia, lung cancer, multiple myeloma, neuroendocrine cancer, small cell lung cancer (SCLC), TAZ/WWTR1, YAP

## Abstract

Cancers have traditionally been classified based on their tissue of origin. However, with advances in sophisticated genome sequencing techniques and progression toward an era of precision medicine, it has become increasingly clear that classifying tumors based on unifying molecular features instead of tissue of origin may hold the key to improving patient outcomes. Various efforts have been undertaken to address this critical aspect of cancer biology, but it is still unclear as to the best approach to stratify tumors into different molecular classes. One approach is to define many small subclasses based on complex molecular signatures, while another option is to divide cancers into larger groups based on higher-order features of cancer behavior. This latter approach holds appeal as it may provide opportunities to identify broadly relevant therapeutics. However, our understanding of these fundamental ‘rules’ of cancer biology and how they can be used to better classify and treat cancers is in its infancy. We recently demonstrated that cancers can be functionally stratified into binary YAP^on^ and YAP^off^ super-classes with unique therapeutic vulnerabilities based on distinct expression and function of the transcriptional coactivators, YAP and TAZ. In YAP^on^ cancers, YAP and TAZ drive oncogenesis, whereas in YAP^off^ cancers, YAP and TAZ are instead tumor suppressors. In this review, we discuss our understanding of these distinct cancer classes with a focus on the mechanisms that underlie the opposite function of YAP/TAZ in YAP^on^ and YAP^off^ cancers, as well as the potential therapeutic implications of these findings.

## Introduction

Cancer is a highly complex disease with distinct combinations of mutations, chromosomal abnormalities, and epigenetic alterations making essentially every tumor unique. This is further complicated by clonal evolution and heterogeneity within each tumor. This complexity raises the question as to what is the best way to classify, and ultimately treat, each tumor. Traditionally, tumors have been classified based on their tissue of origin, which in some instances is highly relevant, such as in the case of subsets of breast and prostate cancer. However, in the era of precision medicine and targeted therapies, it is becoming increasingly clear that stratifying tumors based on unique molecular features may hold the key to overcoming the inherent complexity of cancer. Thus, massive genomic and proteomic efforts have been undertaken to define these key molecular features and identify strategies to better classify tumors [1–7]. While great progress has been made in this pursuit, it is still unclear as to the best approach to classify tumors, and how these refined classification systems can be used to pinpoint specific vulnerabilities of each group. One strategy is to use complex molecular signatures to define smaller and smaller cancer subgroups [3,4,8–11]. Alternatively, it may be possible to define larger cancer classes based on overarching rules of cancer behavior to expose broadly relevant therapeutics. For example, there may be therapeutic potential in stratifying tumors based on different *p53* mutations [12], and tumors can be classified as homologous recombination proficient or deficient, with the latter group exhibiting sensitivity to PARP inhibitors [13]. However, our understanding of these fundamental rules of cancer behavior, and the extent to which cancers can be simplified, is still in its infancy. We recently demonstrated that cancers can be functionally stratified into just two types based on distinct expression and function of the transcriptional co-activators, YAP (YAP1) and TAZ (WWTR1) [14,15]. We termed these classes either ‘YAP^on^’ or ‘YAP^off^’. In this review, we discuss these two cancer classes with a focus on their distinct molecular features and the potential therapeutic implications of this unique binary cancer classification.

## Stratification of cancers into binary YAP^on^ and YAP^off^ classes

**Figure 1:**
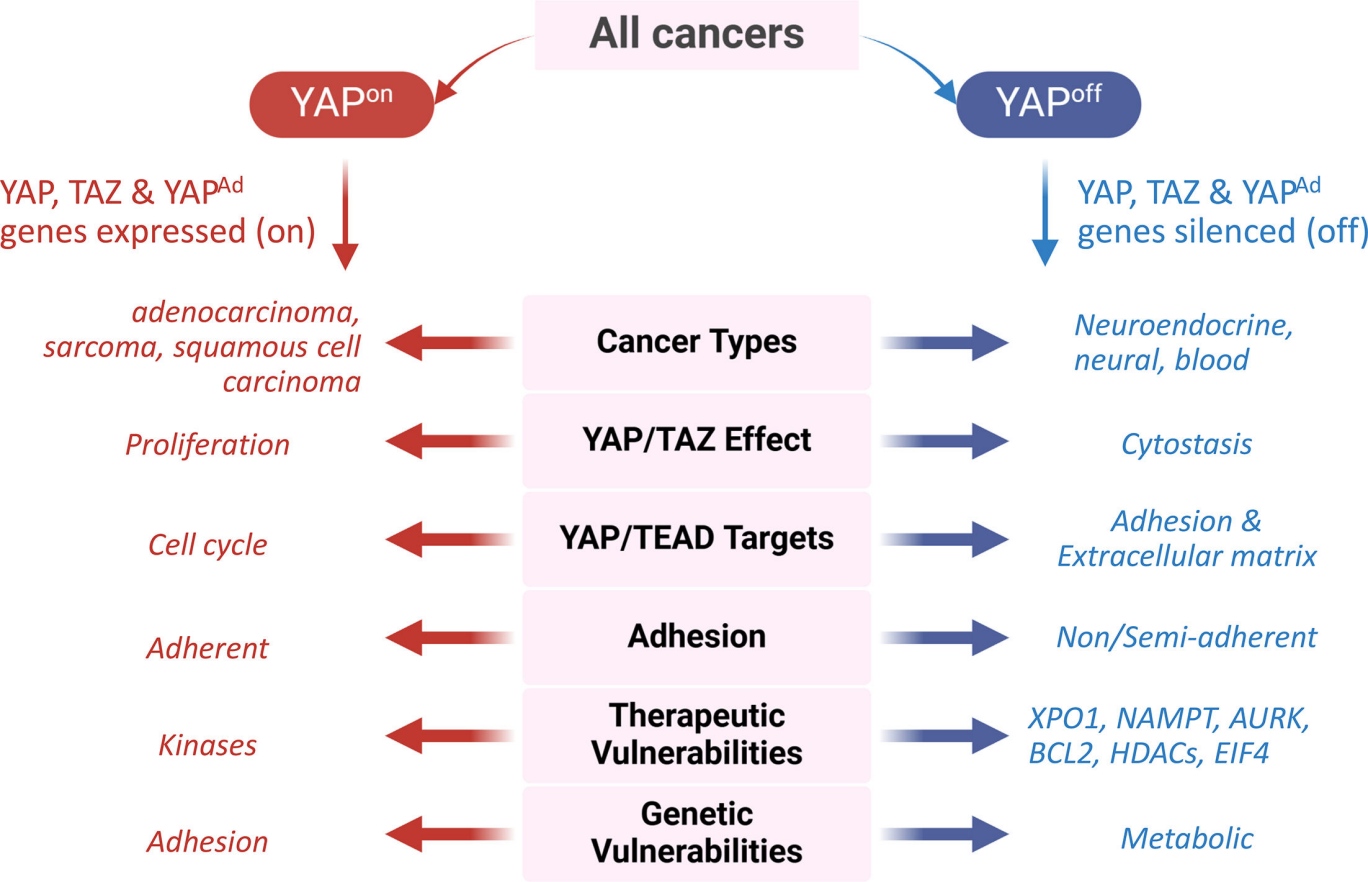
Defining features of YAP^on^ and YAP^off^ cancers. Cancers can be functionally stratified into binary YAP^on^ and YAP^off^ cancer classes based key transcriptomic and functional differences. YAP^Ad^, YAP adhesion target genes.

Using an unbiased approach, we previously demonstrated that cancers can be functionally stratified into two fundamentally distinct classes based on expression of the transcriptional co-activator YAP, its paralog TAZ, and a set of ~80 of their adhesion-related target genes [14,15] (Figure 1). In one class, termed YAP^on^, YAP, TAZ, and their adhesion targets are expressed, whereas in the other class, termed YAP^off^, these genes are instead epigenetically silenced [14,15]. We initially termed this class-defining gene signature (*YAP*, *TAZ,* and their adhesion targets) ‘PC1^+^’ in reference to the fact that it was identified using principal component analysis [14], but we now refer to these as YAP^Ad^ genes, for YAP adhesion target genes. YAP^off^ cancers consist of almost all hematological, most neuroendocrine (NE), and many neural cancers from various tissues, including cancers such as small cell lung cancer (SCLC), NE prostate cancer (NEPC), Merkel cell carcinoma (MCC), retinoblastoma, and subsets of neuroblastoma and medulloblastoma, among many others [14]. YAP^off^ cancers can be further subdivided into liquid (hematological) and solid (NE and neural) YAP^off^ cancers, based on a second gene signature consisting of antigen processing and presentation (high in liquid YAP^off^ cancers) and NE/neural genes (high in solid YAP^off^ cancers) [14]. In contrast, YAP^on^ cancers consist of other solid tumors, such as sarcomas, adenocarcinomas, and squamous cell carcinomas from various tissues [14]. These can also be divided into two subgroups based on the same antigen processing/presentation and NE/neural gene signature.

Despite the YAP^on^/YAP^off^ nomenclature, which was adopted because YAP is a top-scoring YAP^Ad^ gene and a key regulator of the YAP^Ad^ gene signature [14], it is important to note that the expression of YAP is not the sole defining feature of YAP^on^ or YAP^off^ cancers. Cancers can therefore be classified as YAP^on^ without expressing YAP. Instead, YAP^on^ and YAP^off^ cancers are defined by opposite expression of the YAP^Ad^ gene signature—high (‘on’) in YAP^on^ cancers and low (‘off’) in YAP^off^ cancers—along with key functional and phenotypic differences (Figure 1). One of the major defining features of these cancer classes is the distinct function of YAP and TAZ in each class. In YAP^on^ cancers, YAP and TAZ are oncogenes, whereas in YAP^off^ cancers, they have the opposite effect and function as tumor suppressors [14]. This is discussed in detail below. Beyond this, YAP^on^ and YAP^off^ cancers exhibit distinct adhesive qualities when grown in culture; YAP^on^ cancers grow as adherent cultures with epithelial or mesenchymal morphology, whereas YAP^off^ cancers are non- or semi-adherent and typically exhibit a rounded morphology [14]. Importantly, YAP^on^ and YAP^off^ cancers also exhibit distinct therapeutic and genetic vulnerabilities, highlighting the potential clinical implications of this binary classification system [14]. Thus, YAP^on^ and YAP^off^ cancers are not just defined by a gene signature but exhibit profound functional and phenotypic differences with important clinical implications. Despite this, many critical questions must still be addressed to better understand these two fundamentally distinct cancer classes and their therapeutic implications. At the forefront is delivering a better understanding of the molecular basis of these profound differences—why do YAP and TAZ have opposite roles in YAP^on^ and YAP^off^ cancers—and how we can exploit these differences to better treat patients?

## The role of YAP and TAZ in YAP^off^ cancers

To understand the molecular basis of YAP^on^/YAP^off^ cancers, it is critical to understand one of the core fundamental differences between these cancer classes, which is the distinct function of YAP/TAZ in each class [14,15]. In YAP^on^ cancers, YAP and TAZ have well-characterized pro-oncogenic roles, whereas YAP/TAZ exert a potent tumor suppressor function in YAP^off^ cancers. In many YAP^on^ cancers, high YAP and TAZ activity is associated with poor patient outcomes [16–18]. In this regard, YAP/TAZ exert their pro-oncogenic role through various mechanisms, such as promoting cancer cell proliferation, survival, stemness, plasticity, drug resistance, invasion, metastasis, angiogenesis, and immune evasion [16–18]. This classic, pro-oncogenic role of YAP and TAZ in YAP^on^ cancers has been thoroughly reviewed elsewhere [16–18], so here we will primarily focus on the anti-cancer function of YAP/TAZ in YAP^off^ cancers, which has now been demonstrated by many groups.

### Inhibition of cell proliferation and survival

**Figure 2:**
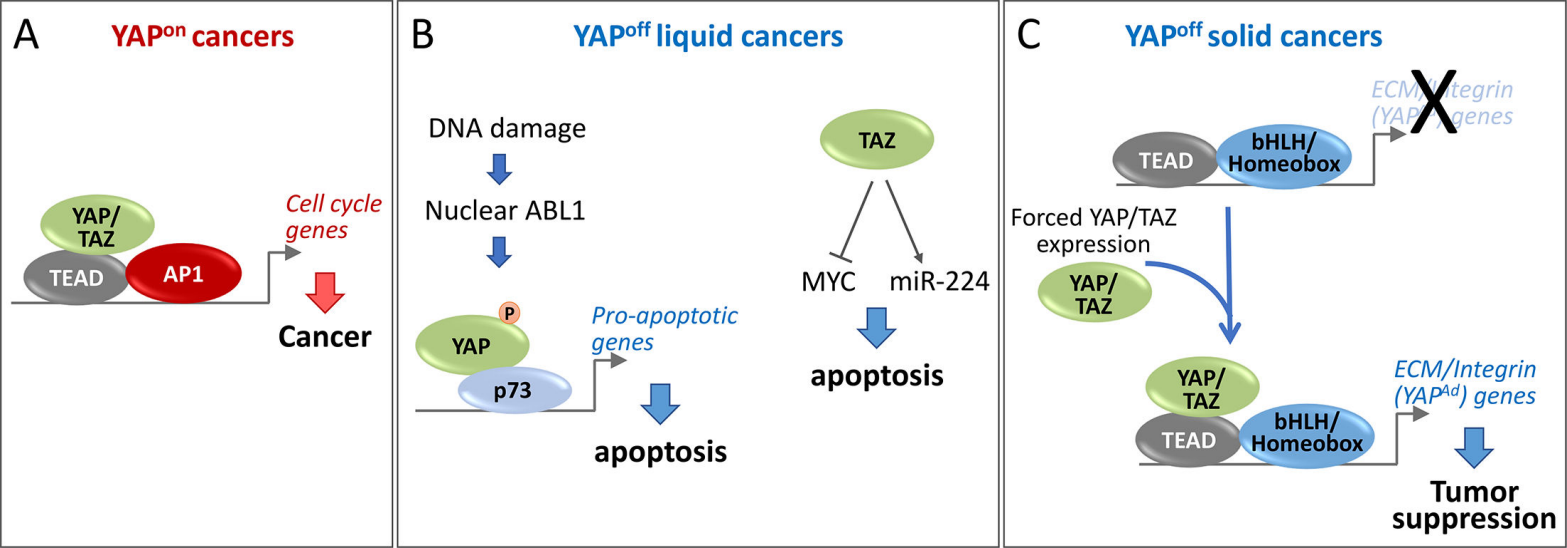
Mechanisms underlying distinct YAP/TAZ function in YAP^on^ and YAP^off^ cancers. **(A**) In YAP^on^ cancers, YAP/TAZ co-operate with TEADs and activator protein 1 (AP1) transcription factors to induce expression of cell cycle genes and promote cancer growth. (**B** ) In YAP^off^ liquid cancers, forced expression of YAP co-operates with p73 to up-regulate pro-apoptotic genes, causing apoptosis. Alternatively, forced TAZ expression down-regulates MYC and up-regulates miR-224 to induce apoptosis. (**C** ) In YAP^off^ solid cancers, forced YAP/TAZ co-operate with TEADs to induce extracellular matrix (ECM) and adhesion (YAP^Ad^) genes causing cytostasis, apoptosis, and reduced invasion. These effects of YAP/TAZ-TEAD may involve co-operation with basic helix-loop-helix (bHLH) and/or homeobox transcription factors, although this remains to be determined. YAP^Ad^, YAP adhesion target genes.

YAP and TAZ are silenced in YAP^off^ cancers. Therefore, their anti-cancer function in this context has largely been revealed by gain-of-function studies where YAP or TAZ are ectopically expressed in these cells, although these findings have been supported by several loss-of-function studies. In cell lines from many solid YAP^off^ cancers, including SCLC [14,19–21], retinoblastoma [14,21,22], NEPC [14], NE pancreatic cancer [14], NE breast [14], lung carcinoid [23], and MCC [24], forced YAP and/or TAZ expression impairs cell growth. Some studies demonstrated that this is primarily due to reduced proliferation, in particular S-phase blockade [14,24], whereas others noted reduced survival following ectopic YAP expression [19,22], suggesting that YAP/TAZ may block growth of YAP^off^ solid cancers through both mechanisms. Whether this exact mechanism differs by cancer type remains to be determined. Induction of a doxycycline-inducible *Yap* transgene also reduced SCLC proliferation and tumorigenesis *in vivo* [14], confirming *in vitro* studies. YAP and TAZ are transcriptional co-activators but do not possess DNA-binding ability [25]. Thus, they must be recruited to specific target genes by DNA-binding transcription factors [25]. Several different YAP/TAZ partners have been identified, but the pro-oncogenic activity of YAP/TAZ in YAP^on^ cancers is primarily mediated via the TEAD-family (TEAD1-4) of DNA-binding proteins [26–29]. Strikingly, the anti-cancer activity of YAP/TAZ in solid YAP^off^ cancers is also dependent on TEADs [14,24], further highlighting the dichotomy between these cancer classes (Figure 2). Together, these studies demonstrate that silencing of *YAP* and *TAZ* is obligatory across many YAP^off^ solid cancers, although one group did not observe any obvious growth defects when YAP was ectopically expressed in SCLC cell lines, but did observe effects on cell adhesion and migration (discussed more below) [30]. Why this study [30] did not recapitulate the growth-inhibitory effects observed by several other groups [14,19–21] is unknown, but it could be due to differences in YAP expression levels or cell line-specific effects.

Complementing these gain-of-function approaches, loss-of-function studies in SCLC and retinoblastoma have further demonstrated an anti-cancer role for YAP/TAZ [14,31]. Unlike human retinoblastoma, which lacks YAP and TAZ expression [14,21,22], murine retinoblastoma expresses Yap/Taz [14]. Strikingly, knockout of *Yap* or *Taz* dramatically increased tumorigenesis of these low-penetrance tumors, which was further enhanced when both *Yap* and *Taz* were deleted [14]. Additionally, knockout of *Yap* and *Taz* in murine *Rb1/p53*-null SCLC promoted tumor initiation, although long-term effects were not assessed [14]. This latter result was somewhat surprising given that lung NE cells, a primary cell-of-origin of SCLC [32], lack Yap/Taz expression [14,20,33]. However, SCLC can also arise from lineages beyond lung NE cells, such as basal cells [34,35] as well as alveolar type II cells at low frequency [32,34]. This suggests the possibility that knockout of *Yap/Taz* in these alternative lineages may promote transformation to SCLC, which is consistent with the role of YAP/TAZ in counteracting the NE lineage [20,23,33]. In contrast, another group reported that knockout of *Yap* and *Taz* in *Rb1/p53/Pten*-null SCLC reduced tumor initiation [20]. The reason for these discrepant results is unknown but may be related to the inclusion of *Pten* knockout in the latter model [20]. Alternatively, it may be due to different roles of Yap/Taz in different SCLC cells-of-origin, as this latter model initiated SCLC specifically from lung NE cells using a NE-restricted Cre driver [20], whereas other work targeted a wider range of cell types using a broadly expressed Cre [14]. Beyond these *in vivo* studies, an *in vitro* positive selection screen identified YAP and TAZ as key mediators of cell death caused by combined LSD1/KDM5 inhibition in SCLC [31]. In this context, LSD1/KDM5 inhibition resulted in up-regulation of YAP and TAZ expression across multiple SCLC lines, and YAP or TAZ knockout partially rescued LSD1/KDM5-induced cell death in several of these lines [31]. This suggests that YAP/TAZ induction contributed to cell death following LSD1/KDM5 inhibition, consistent with an anti-cancer function in YAP^off^ cancers. Together, these extensive *in vitro* and *in vivo* studies across multiple cancer contexts have established a definitive role for YAP and TAZ in inhibiting proliferation, survival, and tumorigenesis of YAP^off^ solid cancers, which is in stark contrast to the pro-proliferation and pro-survival roles of YAP/TAZ in YAP^on^ cancers [14,16,17].

Similar to YAP^off^ solid cancers, YAP and TAZ also exhibit an anti-oncogenic function in YAP^off^ liquid cancers [36–41]; however, the mechanisms are likely distinct from those observed for YAP^off^ solid cancers (Figure 2). In multiple myeloma and leukemia cell lines, forced expression of YAP drives p73-dependent apoptosis, and in a rare multiple myeloma line that expressed YAP, knockdown of YAP increased survival [36]. Similarly, knockdown or inhibition of negative regulators of YAP (e.g., the MST1 kinase) results in YAP induction and YAP-dependent death of multiple myeloma and leukemia lines [36,37]. Mechanistically, this is due to high levels of DNA damage, which drives nuclear accumulation of the ABL1 kinase, which phosphorylates YAP to promote YAP/p73-dependent cell death [36,37]. Furthermore, forced or induced TAZ expression also causes apoptosis of multiple myeloma cell lines [38–41]. Interestingly, these effects of TAZ are likely independent of binding to TEADs or p73, suggesting a distinct mechanism from that observed in YAP^off^ solid cancers [14,24] and from the mechanism of YAP in multiple myeloma [36,37]. This activity of TAZ in multiple myeloma is likely due, at least in part, to down-regulation of MYC [38] and induction of miR-224 [40], albeit through unknown mechanisms. It was also reported that *Yap* and *Taz* are dispensable for leukemogenesis, since their genetic knockout did not alter transformation in multiple acute myeloid leukemia (AML) models [42]. Although, given that YAP/TAZ are typically silenced in AML [14,36], this is not overly surprising. Together with the work in YAP^off^ solid neural and NE cancers, this work in blood cancers demonstrates that silencing of YAP and TAZ is essential for the survival and/or proliferation of most, if not all, YAP^off^ cancers.

### Counteracting cell fate, morphology, and metastasis

Beyond their role in proliferation and survival, YAP and TAZ activity have a profound influence on tumor cell fate, adhesion, and metastasis. Most strikingly, YAP and TAZ expression predicts adhesive vs. non-adhesive growth characteristics *in vitro*—YAP^on^ cells exhibit an epithelial or mesenchymal morphology and grow as adherent cultures, whereas YAP^off^ cells are rounded and grow as non- or semi-adherent cultures [14,20,24,33,43] (Figure 1). These growth characteristics are consistent with the silencing of many adhesion and extracellular matrix YAP^Ad^ genes in YAP^off^ cancers [14]. Importantly, forced expression of YAP in YAP^off^ cancers induces the expression of these class-defining YAP^Ad^ genes [14,23,24]. Furthermore, ectopic expression of YAP alters cellular morphology and adhesion by driving YAP^off^ cells toward an epithelial/mesenchymal, adherent morphology [14,20,30,33] or causing them to form more tightly packed clusters [24]. Knockdown of YAP in rare YAP^on^ SCLC cell lines also causes cells to adopt a more rounded morphology [43]. Consistent with these morphological changes, ectopic YAP expression dramatically reduces metastatic capabilities of SCLC by inhibiting their aggressive amoeboid-like movement, likely through induction of the classic YAP/TAZ target genes, *CYR61* and *CTGF* [30]. Thus, YAP silencing is required for the highly metastatic capabilities of SCLC [30] and potentially other small cell NE YAP^off^ cancers. A rounded, non-/semi-adherent morphology is a hallmark of YAP^off^ NE cancers [14,20,24,30,33]. Consistent with YAP counteracting this NE morphology, YAP also counteracts the NE fate of many of these tumors, as forced YAP expression causes a down-regulation of NE markers in SCLC [20,33], lung carcinoid [23], and NEPC [44]. Thus, YAP/TAZ play a key role in controlling cell fate and are master regulators of the class-defining YAP^Ad^ genes that stratify binary YAP^on^/YAP^off^ cancers as well as the distinct cellular morphology that typifies YAP^off^ cancers.

## Mechanisms underlying distinct YAP/TAZ function in YAP^off^ cancers

Work from many groups definitively demonstrates that YAP and TAZ have distinct functions in YAP^on^ and YAP^off^ cancers, which is a key defining feature of these binary classes [14,15]. However, a fundamental question that remains to be fully addressed is *why* do YAP/TAZ have opposite functions in these two cancer classes—why do they drive cytostasis and cell death in YAP^off^ cancers, while driving proliferation and survival in YAP^on^ cancers?

In YAP^on^ cancers, YAP/TAZ co-operate with many different transcription factors, including RUNX, SMAD, ERBB4, and p63/p73 [16,45], although the primary mediators of YAP/TAZ-driven oncogenesis are TEAD-family DNA-binding proteins [26–29]. Specifically, TEADs recruit YAP/TAZ to distal enhancers of cell cycle and invasion genes where they co-operate with activator protein 1 (AP1)-family transcription factors to drive expression of these pro-oncogenic processes [46,47] (Figure 2A), likely through the recruitment of B-MYB to the Myb-MuvB complex [48]. In YAP^off^ blood cancers, YAP drives apoptosis independent of TEADs, primarily through a p73-dependent mechanism [36] (Figure 2B). It was demonstrated that high levels of DNA damage in blood cancer lines drive nuclear accumulation of ABL1, which in turn phosphorylates YAP on tyrosine 357 [36], a modification that promotes association of YAP with p73 and reduces transcriptional activity of YAP/TEAD complexes [49,50]. Thus, this unique cellular context, exemplified by high levels of DNA damage [36], may explain the function of YAP in YAP^off^ blood cancers. However, TAZ is not believed to bind p73 [45], and the anti-cancer role of TAZ in multiple myeloma is likely independent of p73 and TEAD, since mutating the TEAD binding site or WW domain (WW domains mediate YAP/p73 interaction [51]) of TAZ does not affect TAZ function in this context [38]. This suggests a distinct mechanism of action for TAZ in blood cancers, and indeed, it was shown that TAZ exerts its anti-cancer activity via down-regulation of MYC [38] and induction of miR-224 [40] in multiple myeloma (Figure 2B). The exact mechanisms that underlie this unique function of TAZ in multiple myeloma and the partners it co-operates with, if any, are unclear but warrant further investigation. Despite this, the anti-cancer activity of YAP/TAZ in YAP^off^ liquid cancers can largely be explained by TEAD-independent mechanisms, which are, at least in part, due to the unique cellular context of these tumors (e.g., high nuclear ABL1 levels [36]).

Unlike in YAP^off^ blood cancers, the tumor suppressor function of YAP and TAZ in YAP^off^ solid cancers is dependent on TEADs (Figure 2C) [14,24,30] the same mediators of YAP/TAZ oncogenic activity in YAP^on^ cancers (Figure 2A) [26–29]. Multiple lines of evidence support a key role for TEADs in this context: (1) mutating the TEAD-binding site in YAP or TAZ completely blocks their anti-cancer activity in multiple YAP^off^ solid cancers [14, 24, 30], (2) knockout of TEADs abrogates, while overexpression of TEADs enhances, YAP-driven cytostasis in SCLC [14], and (3) a TEAD DNA-binding domain fused to a VP64 transcriptional activation domain, which mimics YAP/TEAD activity [28,52], phenocopies the effects of ectopic YAP in YAP^off^ cancers [14]. In addition, mutation of YAP WW domains, which are required for association with p73 [51], does not affect YAP-driven cytostasis in YAP^off^ solid cancers [14]. This argues that the YAP/p73-dependent mechanism identified in some blood cancers [36] is not at play in YAP^off^ solid cancers, further supporting a role for YAP/TEAD in these cancers. Thus, the YAP/TAZ-TEAD complex has opposite functions in YAP^on^ and YAP^off^ solid cancers.

In YAP^on^ cancers, YAP/TAZ-TEAD promote expression of cell cycle genes [14,16–18,46,48]; however, in YAP^off^ solid cancers, ectopic YAP does not induce cell cycle genes and instead up-regulates extracellular matrix, cytoskeletal, and adhesion genes (e.g., YAP^Ad^ genes) [14,23,24,30]. Interestingly, in SCLC and retinoblastoma, YAP/TEAD-driven cytostasis is partially mediated via induction of an integrin-αV/β5-Netrin1-UNC5B axis [14,21], whereas down-regulation of Merkel cell polyomavirus large T antigen, likely through indirect mechanisms, is involved in suppressing polyomavirus-positive MCC [24]. While this latter mechanism would be specific to this subset of MCC, it is unknown whether the integrin/Netrin axis is relevant to other YAP^off^ cancers or whether distinct pathways are involved in different YAP^off^ solid cancers. Of further note, blocking integrin-αV/β5-Netrin1-UNC5B activity only partially rescues cell growth, demonstrating that additional mechanisms are involved. Future work across a wide range of YAP^off^ solid cancers is required to deduce these important mechanisms. However, regardless of the specific downstream targets, it is clear that distinct YAP/TAZ-TEAD transcriptional programs underlie opposite YAP/TAZ function in YAP^on^ and YAP^off^ solid cancers. To better understand why the YAP/TEAD complex regulates distinct genes in these cancer classes, we performed TEAD ChIP-Seq across SCLC and retinoblastoma cell lines [14]. Strikingly, comparison of these data to published TEAD ChIP-Seq from various YAP^on^ cancers demonstrated distinct binding patterns and showed that TEADs (and presumably YAP/TAZ) are recruited to distinct enhancers in YAP^on^ vs. YAP^off^ solid cancers [14]. Thus, this argues that distinct genomic targeting probably underlies unique YAP/TEAD target gene repertoires and function in YAP^on^ vs. YAP^off^ cancers.

The next question this raises is why YAP/TEAD are recruited to distinct loci in each cancer class? One possibility is that YAP/TEAD co-operate with different partners that guide YAP/TEAD to unique genomic loci in each class. Motif and transcription factor co-binding analysis of TEAD-binding sites in YAP^on^ and YAP^off^ cancers demonstrated a strong enrichment for AP1-family transcription factors in YAP^on^ cancers [14], consistent with the known co-operation of YAP/TEAD/AP1 in these cancers [46,47]. In contrast, YAP^off^-specific TEAD sites were not enriched for AP1 motifs and were instead enriched for neurogenic basic helix-loop-helix (bHLH) and homeobox transcription factors [14]. This observation supports the possibility that distinct partner proteins may influence the opposite YAP/TEAD function in YAP^on^ and YAP^off^ solid cancers (Figure 2C), although this remains to be tested. Another possibility is that each class exhibits a distinct chromatin landscape, which ultimately changes accessible loci for YAP/TEAD complexes. Indeed, many YAP^off^ NE cancers exhibit profoundly different regions of chromatin accessibility compared with YAP^on^ adenocarcinomas [53]. Not surprisingly, uniquely open regions in NE YAP^off^ cancers are highly enriched for pathways involved in neural development and binding sites for classic neural/NE bHLH transcription factors, such as ASCL1 and NEUROD1 [53]. Furthermore, in an experimental model where tumor cells convert from YAP^on^ adenocarcinomas to YAP^off^ NE cancer, cells undergo major chromatin remodeling with over 10,000 genomic loci changing in their accessibility [54]. In this model, sites for classic neural/NE transcription factors become hyperaccessible in YAP^off^ solid tumors, whereas TEAD binding sites become hypoaccessible in YAP^off^ cells [54]. Furthermore, in human patients, binding sites for AP1 transcription factors, key YAP/TEAD partners in YAP^on^ cancers [14,46,47], are the top hypermethylated transcription factor motif in SCLC (YAP^off^) compared with lung adenocarcinoma (YAP^on^) [55], and in mouse models, AP1 sites were hyperaccessible in lung adenocarcinoma compared with SCLC [56]. Thus, YAP^on^ and YAP^off^ cancers exhibit dramatically different chromatin landscapes, and many silenced genomic loci in YAP^off^ cancers map to YAP/TEAD/AP1 binding sites, supporting the notion that distinct chromatin landscapes may influence differing YAP/TEAD activity. However, this is somewhat paradoxical, given that key YAP/TEAD/AP1 sites in YAP^on^ cancers map to enhancers for many core cell cycle genes [46]. It is therefore possible that these cell cycle genes are regulated by distinct mechanisms in YAP^off^ cancers, such as high activity of MYC-family transcription factors, which typifies both solid and liquid YAP^off^ cancers [14], or high E2F activity which is common in many YAP^off^ solid cancers due to high frequency of *RB1* gene loss [14]. It is also possible that these two mechanisms are not mutually exclusive, given that many binding sites that open in YAP^off^ cancers (e.g., neurogenic bHLH factors) match putative TEAD partners in these cancers [14]. Future work in this area will be critical to explore the contribution of these, and other, mechanisms to the opposite functions of YAP/TAZ-TEAD in YAP^on^ vs. YAP^off^ solid cancers.

## Mechanisms mediating silencing of *YAP* and *TAZ* in YAP^off^ cancers

**Figure 3:**
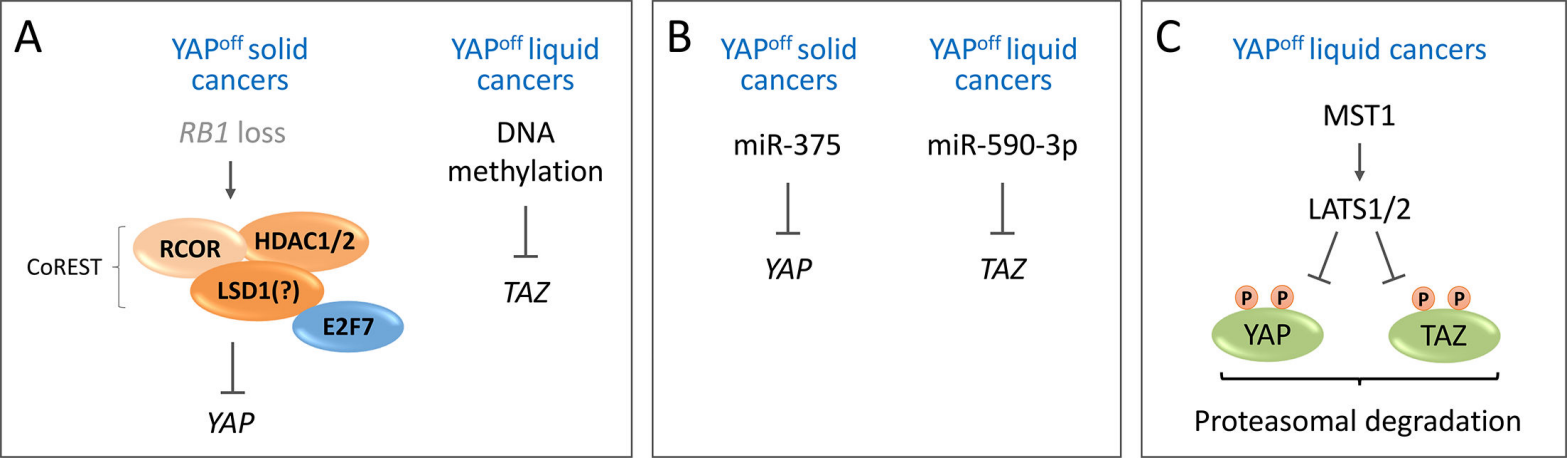
Mechanisms mediating YAP and TAZ silencing in YAP^off^ cancers. (**A**) Chromatin modifications mediating *YAP/TAZ* silencing. In YAP^on^ solid cancers, *RB1* loss results in E2F7 up-regulation, which in turn recruits the CoREST complex to silence the *YAP* promoter. However, the role of LSD1, a core CoREST component, may vary between different samples. In YAP^off^ liquid cancers DNA methylation plays a role in TAZ silencing. (**B** ) MicroRNAs (miRNAs) that mediate YAP/TAZ down-regulation. In YAP^off^ solid cancers, miR-375 may contribute to YAP down-regulation, whereas miR-590–3p regulates TAZ expression in YAP^off^ liquid cancers. (**C** ) In YAP^off^ liquid cancers, the Hippo pathway (MST/LATS kinases) plays a role in maintaining YAP and TAZ down-regulation by targeting YAP and TAZ for proteasomal degradation.

Another fundamental aspect in understanding the molecular basis of YAP^on^ and YAP^off^ cancers is determining what controls expression of the YAP^Ad^ genes that define these cancer classes, more specifically, what mediates their silencing in YAP^off^ cancers [14,15]. YAP and TAZ are both top-ranking YAP^Ad^ genes, and when ectopically expressed in YAP^off^ cancers, they induce expression of this class-defining gene signature [14], demonstrating that YAP/TAZ are master regulators of this signature. Therefore, mechanisms that mediate *YAP* and *TAZ* silencing in YAP^off^ cancers are key to maintaining silencing of the entire YAP^Ad^ signature that defines these cancers. While there are rare instances of deletion/mutation of *YAP* in YAP^off^ cancers, for example, ~ 10% of multiple myeloma possess *YAP* deletions [36], *YAP* and *TAZ* are not commonly deleted in cancer [57], so this does not appear to be the primary mechanism of *YAP/TAZ* silencing in YAP^off^ cancers. Instead, *YAP*, *TAZ,* and the YAP^Ad^ genes are primarily silenced at the epigenetic level [14], although in certain contexts, additional mechanisms may also contribute, as discussed below (Figure 3).

### Epigenetic mechanisms mediating *YAP/TAZ* gene silencing in YAP^off^ cancers

#### Chromatin modifications

Various epigenetic mechanisms appear to contribute to silencing of *YAP/TAZ* in YAP^off^ cancers (Figure 3A), although these may vary depending on the specific cancer or across different samples from the same cancer type. For example, the LSD1 (KDM1A) histone demethylase has been implicated in *YAP/TAZ* silencing in certain contexts. LSD1 is up-regulated in multiple YAP^off^ cancers [58–60], and LSD1 inhibitors show strong, although sometimes variable, efficacy against these cancers [58–64] and are currently being tested in clinical trials for SCLC and other NE cancers [65]. Interestingly, in a panel of six SCLC cell lines treated with the LSD1 inhibitor, ORY-1001, YAP and TAZ were significantly induced in two or four of the lines, respectively [31]. Furthermore, in a panel of SCLC patient-derived xenografts treated with combined LSD1 and KDM5 inhibitors, YAP and/or TAZ were induced in five of seven samples [31,62]. A recent pre-print also demonstrated that LSD1 silences YAP in NEPC [66]. However, another group found that treatment of two separate SCLC lines with an LSD1 inhibitor did not affect YAP expression [30]. Thus, LSD1-mediated histone methylation may be involved in silencing YAP/TAZ in some instances of SCLC and other YAP^off^ solid cancers. In addition to SCLC, LSD1 inhibitors are being tested in clinical trials for AML [65], a YAP^off^ blood cancer [14]. LSD1 could also contribute to YAP/TAZ silencing in YAP^off^ blood cancers, although this requires further testing.

Histone deacetylases (HDACs) are a large family of transcriptional repressors that also contribute to *YAP/TAZ* silencing in some YAP^off^ cancers. For example, treatment of the NCI-H660 NEPC cell line with the pan-HDAC inhibitor, Vorinostat, resulted in induction of YAP [44], and treatment of multiple myeloma cell lines with pan-HDAC inhibitors caused TAZ up-regulation [38]. In contrast, Wu et al. found that treatment with a pan-HDAC inhibitor did not alter YAP expression in several SCLC lines, whereas treatment with Entinostat, a benzamide-family HDAC inhibitor that targets the class I subset of HDACs, resulted in YAP induction [30]. HDACs are components of many epigenetic complexes, but Wu et al. found that it was specifically the CoREST repressive complex responsible for silencing of YAP in these cells since knockout of the core CoREST components RCOR1/2/3 similarly induced YAP expression [30]. It is also noteworthy that LSD1 is a core component of the CoREST complex [67,68], although in the work by Wu et al., LSD1 did not appear to have a role in silencing YAP expression [30]. Interestingly, in this context, induction of YAP required the SIN3-HDAC complex [30], and it was argued that this explained why the pan-HDAC inhibitors did not alter YAP expression, as benzamide-family HDAC inhibitors do not efficiently target the SIN3-HDAC complex [30]. This suggests that the regulation of YAP in these SCLC cell lines may be distinct from that of the H660 NEPC line [44], although work on a wider range of models from these, and other YAP^off^ cancers, is required to deduce if these differences represent variability between different YAP^off^ cancers, or simply sample-to-sample variability. Wu et al. further went on to demonstrate that *RB1* loss in SCLC resulted in up-regulation of E2F7, which in turn recruited CoREST to the promoter of *YAP* to silence it [30]. Whether TAZ was regulated similarly was not assessed. Interestingly, YAP^off^ cancers are highly sensitive to HDAC inhibitors, compared with YAP^on^ cancers [14,69], much like with LSD1 inhibitors [58–64]. This highlights a unique epigenetic landscape that is essential to the survival of YAP^off^ cancers and is also linked to the silencing of *YAP* itself.

In addition to silencing via histone modifications, such as histone methylation and deacetylation, DNA methylation may play a role in TAZ silencing in some contexts [38,41]. Specifically, in multiple myeloma cell lines, inhibition of DNA methyltransferases results in TAZ up-regulation, suggesting that DNA methylation plays a key role in the silencing of *TAZ* [38,41]. Interestingly, in a small number of YAP^off^ NEPC and SCLC lines, DNA methylation doesn’t appear to be a major mechanism of *YAP* silencing since treatment with DNA methyltransferase inhibitors did not alter YAP expression [30,44]. Whether these differences represent distinct mechanisms of regulation of YAP and TAZ (i.e., TAZ is regulated by DNA methylation, whereas YAP is not), differences between cancer types (i.e., multiple myeloma vs. SCLC), or cell line/sample-to-sample variation remains to be determined, particularly since YAP and TAZ regulation were not tested together in these studies. In support of the notion that this may be related to sample-to-sample variability, methylation of the *YAP* or *TAZ* promoters varies in SCLC [44,70] and multiple myeloma [39], respectively. Furthermore, analysis of *YAP* expression and promoter methylation across SCLC cell lines in the SCLC-CellMiner database [71] shows variability in *YAP* promoter methylation and a lack of methylation in many SCLC lines that also lack *YAP* expression. Furthermore, using targeted ChIP-qPCR for *YAP*, *TAZ,* and their target genes *CYR61* and *AJUBA* in a small panel of solid YAP^off^ cell lines representing NE breast, SCLC, and retinoblastoma, we observed significant variability in repressive histone modifications and DNA methylation [14]. While at least one repressive modification was present on the promoters of each gene, these varied between the different genes and cell lines [14]. Importantly, the promoters of these genes lacked activating histone modifications and recruitment of RNA polymerase II, in contrast with YAP^on^ lines [14]. Thus, while it is clear that YAP^off^ cancers must silence *YAP* and *TAZ* and that epigenetic mechanisms play a key role in this, the specific mechanisms may vary across different genes, cancer types, and samples. While reactivating this gene program represents an attractive therapeutic option for YAP^off^ cancers, this approach may be complicated by this variability. Thus, further efforts across a wider range of YAP^off^ cancers will be required to thoroughly define mechanisms of *YAP/TAZ* silencing and identify potential biomarkers for these distinct mechanisms.

#### The role of microRNAs

In addition to the central role that chromatin modifications play in silencing *YAP/TAZ* expression in YAP^off^ cancers, microRNAs (miRNAs) appear to play a key role in some contexts (Figure 3B). For example, miR-375 is a transcriptional target of the NE transcription factor, ASCL1 [19], and is highly expressed in YAP^off^ SCLC [19] and lung carcinoid tumors [23]. Furthermore, miR-375 plays a key role in down-regulating YAP expression and promoting NE differentiation in lung carcinoid cells [23], as forced expression of miR-375 causes down-regulation of YAP and induction of NE genes in YAP^on^ non-small cell lung cancer (NSCLC) cells [19]. Thus, miR-375 contributes to YAP down-regulation in YAP^off^ lung cancers, although whether this mechanism holds true for other YAP^off^ cancers remains to be determined. Interestingly, miR-375 is highly expressed in MCC and promotes NE differentiation in this context [72,73], similar to the lung cancer setting [19,23]. Additionally, high expression of the AATF (CHE-1) transcription factor drives expression of miR-590–3p, which in turn results in TAZ down-regulation in multiple myeloma [41]. Despite this, DNA methylation was shown to play a more prominent role in mediating *TAZ* silencing than miR-590–3p in this context [41], which is consistent with findings that *TAZ* mRNA is dramatically down-regulated in YAP^off^ cancers, including multiple myeloma [14].

### Alternative mechanisms mediating YAP/TAZ down-regulation in YAP^off^ cancers

The lack of *YAP* and *TAZ* mRNA expression in YAP^off^ cancers [14] argues that silencing is primarily mediated via epigenetic mechanisms, as discussed above. However, in multiple myeloma and leukemias, both YAP^off^ blood cancers, YAP [36] and TAZ [39] are also targeted at the protein level via the Hippo signaling pathway (Figure 3C). The Hippo kinase cascade is a key regulator of YAP/TAZ protein expression in many contexts, whereby activation of the MST1/2 and LATS1/2 kinases results in phosphorylation of YAP and TAZ and subsequent proteasomal degradation [74]. Thus, despite a clear role for epigenetic silencing and significant down-regulation at the transcriptional level in YAP^off^ cancers, YAP and TAZ down-regulation is also controlled at the protein level in some YAP^off^ cancers. This highlights a highly complex network responsible for maintaining the silencing of YAP, TAZ, and their class-defining YAP^Ad^ target genes in YAP^off^ cancers. It also demonstrates that while these cancers must turn these factors off, this can be accomplished via different mechanisms. One potential explanation for this could lie in the impact of *RB1* loss, which was shown to drive YAP silencing in SCLC via E2F7 and CoREST [30]. *RB1* is commonly mutated or deleted in SCLC, as well as many other YAP^off^ solid cancers [14,75–77]; however, this is not a common event in YAP^off^ liquid cancers [14], suggesting that mechanisms may differ between these groups.

Additionally, YAP is down-regulated, although not completely silenced, in an *in vitro* model of NSCLC-to-SCLC transformation, which is dependent on the EHMT2 histone methyltransferase [78]. In this context, YAP down-regulation may not be due to direct epigenetic silencing of *YAP* by EHMT2, as it was demonstrated that activation of a WNT/β-catenin pathway downstream of EHMT2 was involved in YAP down-regulation [78]; however, the exact mechanism was not explored. There are several well-established links between the Hippo/YAP and WNT/β-catenin pathways [79–81], although WNT/β-catenin are primarily thought to promote YAP expression/activity in these various YAP^on^ contexts [82–84]. In contrast, YAP can both promote and inhibit WNT/β-catenin signaling, again in the context of a YAP^on^ state [80,85–88]. Thus, the cross-regulation between YAP and WNT/β-catenin is complex and has primarily been studied in YAP^on^ contexts, so whether this is true in a YAP^off^ cancer is unknown. Furthermore, RB1 was also down-regulated downstream of EHMT2-WNT/β-catenin in cells that transform from NSCLC to SCLC [78]. Given that the loss of *RB1* can drive *YAP* silencing [30], it is currently unknown whether RB1 down-regulation, WNT/β-catenin activation, both, or neither pathway is directly involved in YAP down-regulation in this context. This again highlights the complex nature of the mechanisms mediating YAP/TAZ silencing in YAP^off^ cancers.

## Therapeutic implications of YAP^on^/YAP^off^ cancer stratification

### YAP^on^ and YAP^off^ cancers exhibit distinct therapeutic and genetic vulnerabilities

The appeal of identifying novel cancer classification strategies is that they may provide an opportunity to identify novel therapies targeted at the unique biology of different cancer classes. We demonstrated that indeed, YAP^on^ and YAP^off^ cancers exhibit distinct therapeutic and genetic vulnerabilities (Figure 1), suggesting the potential for this classification to guide therapeutic selection. For example, YAP^off^ cancers are highly sensitive to inhibitors of BCL2-family proteins, NAMPT, XPO1, Aurora kinases, and HDACs [14]. These findings are consistent with another pan-cancer study demonstrating that many NE and hematological cancers (YAP^off^ cancers) share vulnerabilities to HDAC, BCL2, and NAMPT inhibitors [69], as well as work showing that NE subsets of SCLC and neuroblastoma are more sensitive to BCL2 inhibitors [89]. Strikingly, when examining genetic vulnerabilities of YAP^off^ cancers, two of the top-scoring hits are *NAMPT* and its partner protein in the NAD + salvage pathway, *NMNAT1*, matching the drug vulnerability data [14]. Beyond these two genes, YAP^off^ cancers exhibit unique dependency on many other metabolic genes, including those of the purine and folate biosynthesis and tricarboxylic acid cycle [14]. Interestingly, many of these genetic dependencies are targets of MYC-family transcription factors, which matches the finding that YAP^off^ cancers exhibit a high frequency of MYC-family amplification and activity [14]. It also suggests that YAP^off^ cancers probably exhibit distinct metabolisms compared with YAP^on^ cancers, which may represent potential therapeutic targets, although this requires further study.

In contrast with YAP^off^ cancers, YAP^on^ cancers exhibit higher sensitivity to various kinase inhibitors, particularly those in the EGFR/MEK/ERK pathway [14,69,89], as well as genetic vulnerabilities to various genes involved in integrin and extracellular matrix signaling and cytoskeletal organization [14]. This latter observation matches the distinct morphological characteristics of YAP^on^ cancers [14]. Beyond this, YAP^on^ cancers are highly dependent on YAP/TAZ [14], a defining characteristic of these cancers. As such, therapeutic strategies to target YAP/TAZ activity hold potential for this cancer class. Various small molecules targeting YAP/TAZ activity have been developed [90], with some targeting the YAP/TEAD interface and others disrupting the interaction of YAP/TAZ with TEADs through inhibiting TEAD auto-palmitoylation activity required for YAP/TAZ-TEAD interaction [91–94]. Thus far, these are undergoing clinical trials for mesothelioma and other NF2 mutant cancers (e.g., NCT04665206 and NCT04857372) [90,94,95], although success against these cancers could spur future studies against various other YAP^on^ cancers. Additionally, a novel dual-targeting CRISPR screening approach developed by Klingbeil et al. identified the MARK2/3 kinases as genetic co-dependencies of YAP and TAZ across a wide range of YAP^on^ cancers, including pancreatic adenocarcinoma, breast, NSCLC, and rhabdomyosarcoma [96]. In contrast, YAP^off^ SCLC and blood cancers were not dependent on MARK2/3 [96]. Mechanistically, it was demonstrated that MARK2/3 phosphorylate and inactivate upstream negative regulators of YAP/TAZ while also phosphorylating YAP/TAZ on positive regulatory sites [96]. Thus, inhibiting MARK2/3 could represent an alternative approach to target YAP/TAZ activity in YAP^on^ cancers, although there are currently no small molecular inhibitors of these kinases [96]. While further work is required to leverage these findings to guide therapeutic selection for YAP^on^ vs. YAP^off^ cancers, these different studies highlight the potential of this approach.

### Implications of YAP^on^/YAP^off^ stratification for immune evasion and immunotherapies

In addition to unique vulnerabilities to small molecular inhibitors, stratification of cancers into YAP^on^/YAP^off^ classes may have implications in immune evasion and the response to immunotherapies. The emergence of immunotherapy has revolutionized cancer treatment by leveraging the immune system to recognize and eliminate tumors [97]. In particular, immune checkpoint inhibitors (ICIs) targeting PD-1, PD-L1, and CTLA-4 have demonstrated remarkable efficacy in several cancers, but their success is variable [98]. A key determinant of response to ICIs is the ability of cancer cells to present antigens through major histocompatibility complex (MHC) molecules, known in humans as the human leukocyte antigen (HLA). Typically, MHC class I (MHC-I)/HLA class I (HLA-I) molecules play a pivotal role in immune surveillance by presenting intracellular peptides on the cell surface. These are then surveyed by cytotoxic CD8+ T cells to recognize and eliminate cells that express non-self-proteins, including viral antigens and tumor-associated antigens [99]. This is accomplished via the antigen processing and presentation machinery, which generates peptides through proteasomal degradation and loads these peptides onto HLA-I molecules for presentation on the cell surface [100]. However, many cancers evade immune control by down-regulating the expression of HLA-I and members of the antigen presentation machinery, which compromises natural immune responses and limits the efficacy of immunotherapies, such as ICIs, which depend on reactivating anti-tumor T cell activity [99]. The loss of HLA-I antigen presentation in cancers can occur through mutation or deletion of structural genes or via alterations in epigenetic, transcriptional, post-transcriptional/pre-translational, or post-translational regulation of HLA-I molecules or other members of the antigen processing and presentation machinery [99]. Epigenetic silencing of HLA-I expression—mediated by DNA methylation, histone modifications, or chromatin remodeling—is a dynamic and reversible process, making this an attractive avenue for therapeutic intervention. For example, epigenetic therapies, such as DNA methyltransferase or HDAC inhibitors, can restore HLA-I expression and enhance immunotherapy efficacy [101,102].

**Figure 4:**
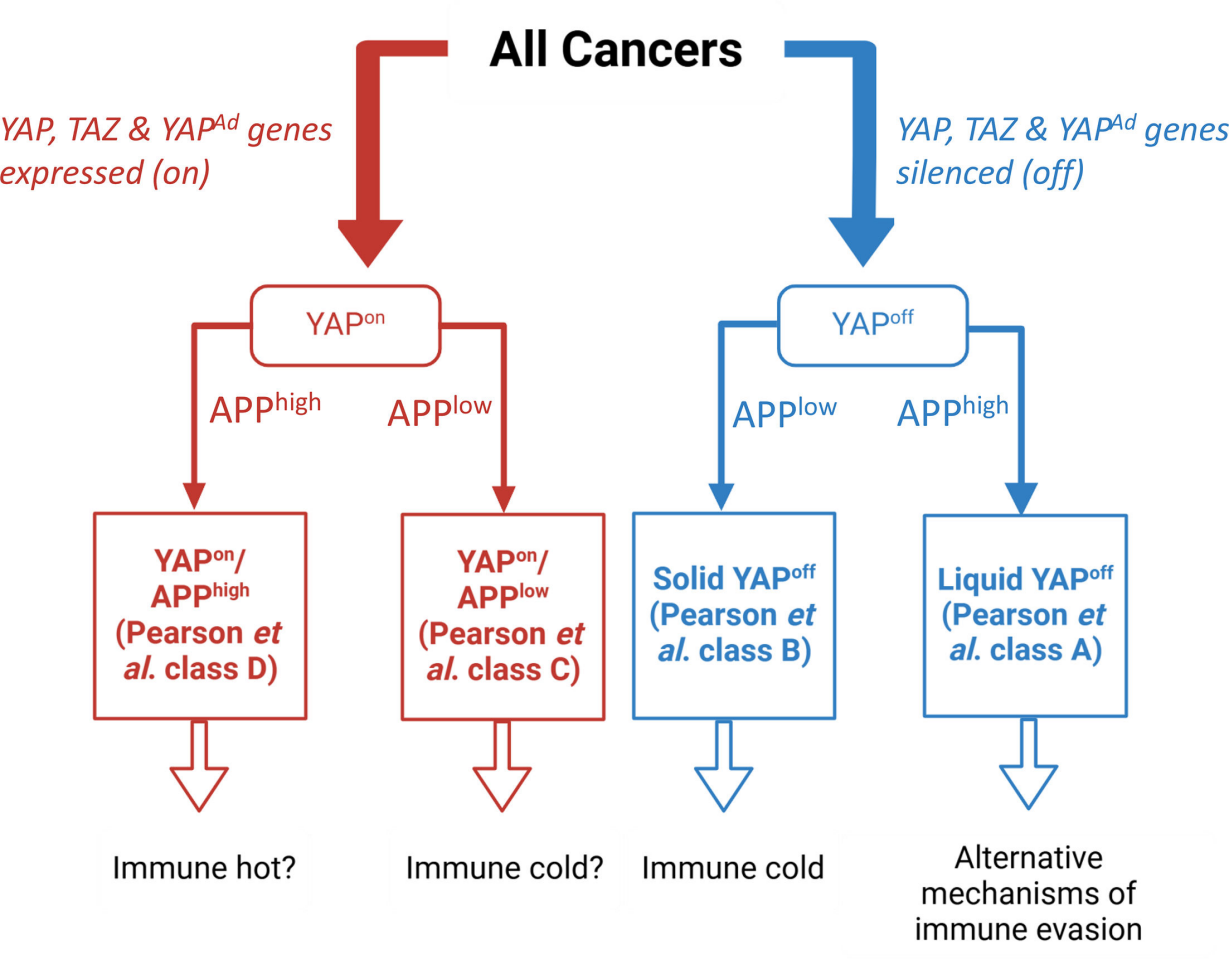
Subclassification of YAP^on^/YAP^off^ cancers based on expression of antigen processing and presentation genes. The YAP^Ad^ gene signature stratifies cancers into YAP^on^ and YAP^off^ super-classes, which can be further divided in four subgroups based on an antigen processing and presentation (APP) gene signature. This latter signature could have implications in immune evasion and response to immunotherapies, although this remains to be formally tested. YAP^Ad^, YAP adhesion target genes.

Interestingly, in our original pan-cancer analysis that stratified cancers into binary YAP^on^ and YAP^off^ classes, cancers could be further divided into four subgroups using a second principle component that consisted of many key antigen processing and presentation genes, including HLA genes themselves (termed the PC3^+^ signature in our original work) [14] (Figure 4). One subgroup is YAP^off^ liquid cancers (e.g., blood cancers), which expressed the highest level of antigen processing and presentation genes [14]. Strikingly, the antigen processing and presentation signature was extremely low in YAP^off^ solid cancers [14]. Consistent with a reduction in antigen presentation, many of these tumors adopt an ‘immune-cold’ phenotype characterized by reduced T cell surveillance [103–106], which is described in detail below. Interestingly, this signature also stratified YAP^on^ cancers into two groups, one with low expression and one with high expression of antigen processing and presentation genes [14]. Thus, understanding HLA expression profiles in YAP^on^ and YAP^off^ tumors may provide insights into their response to immunotherapy. Beyond this, YAP has a direct role in mediating tumor cell immune evasion in some YAP^on^ cancers [16,17], demonstrating that YAP expression is not just correlative, but is functionally important in tumor immune evasion.

#### YAP^off^ cancers and immune evasion

Consistent with our analysis, many YAP^off^ solid cancers are considered immune-cold [103–106]. For example, the majority of NEPC is immune-depleted, with a notable absence of antigen-presenting machinery, including HLA expression, and a lack of infiltrating cytotoxic T cells [103]. Interestingly, however, a small subset of NEPC displays increased inflammatory signatures and immune activity [103]. Whether these may represent a rare YAP^on^ subset of NEPC, similar to observations in SCLC [107] (discussed more below), is an intriguing possibility that warrants further investigation. Interestingly, inflamed NEPC was associated with higher expression of extracellular matrix and integrin genes (e.g., YAP^Ad^ genes) [103], which are also associated with a YAP^on^ state [14], supporting the notion that this subset of inflamed NEPC could represent a rare YAP^on^ subset. Similarly, MCC also shows down-regulation of HLA-I, with 51% of cases in a large retrospective cohort exhibiting a lack of HLA-I expression and another 33% with reduced expression [104]. The silencing of HLA-I in these cells was found to be reversible by stimulating with interferons as well as chemotherapeutic agents [104]. Thus, strategies to restore HLA-I expression in MCC represent a promising treatment path toward improving anti-tumor T cell responses [104].

SCLC is yet another predominantly YAP^off^ cancer that is primarily considered immune cold [105], although ICIs are still part of the standard of care for SCLC despite limited overall efficacy [108–111]. Importantly, recent work demonstrated that a subset of SCLC (~18% of patients) exhibit an inflamed gene signature, and that these are the SCLC tumors that responded to ICIs [112]. Refinement of this inflamed SCLC subtype further stratified it into two subgroups, only one of which benefits from ICIs [113]. Thus, there is a small subset of SCLC tumors that are molecularly distinct from other SCLC and that derive benefit from ICIs. Interestingly, *YAP* mRNA was elevated in the inflamed tumors relative to other SCLC, although there was no difference between the two inflamed subsets [113]. This is somewhat reminiscent of the two YAP^on^ subclasses, although whether this is true remains to be determined. Other groups have also argued that rare patients with YAP-positive SCLC exhibit an inflamed phenotype [114,115] and have shown that YAP-positive SCLC cell lines are more responsive to ICIs *in vitro* [115], potentially linking YAP expression to ICI response in SCLC. Recent work has begun to redefine how SCLC is classified, highlighting at least four molecular subtypes, one of which was proposed to express YAP [107], although the use of YAP as a biomarker for this subtype is controversial [112,114,116,117]. We would argue that these inflamed SCLCs could represent a YAP^on^ state even if they lack YAP expression, although more definitive evidence is required to support this notion.

HLA-I loss in SCLC is primarily epigenetic, allowing potential restoration with epigenetic drugs. Indeed, inhibitors targeting EZH2 or LSD1 enhance HLA-I expression and immune response in preclinical SCLC models [118–120], although clinical efficacy remains unproven. Several clinical trials have explored combination therapies of epigenetic modulators and ICIs in SCLC (e.g., NCT05353439, NCT04631029, NCT06287775, and NCT05191797), but these have faced various challenges, including toxicity and low patient accrual [118,121,122]. Therefore, additional work is required to better define the mechanisms mediating HLA-I silencing in SCLC to identify therapeutic strategies that can be combined to improve ICI efficacy in SCLC. Work linking LSD1 to HLA-I silencing in SCLC [118,119] is also quite intriguing, given that LSD1 contributes to YAP and TAZ silencing in some SCLC [31] (see *Epigenetic mechanisms mediating YAP/TAZ gene silencing in YAP^off^ cancers* section above). Thus, the same mechanisms that control YAP/TAZ silencing may also mediate silencing of antigen processing and presentation genes in SCLC. While future work will be required to deduce the true overlap in these mechanisms, we noted a modest induction of some of these genes when YAP was ectopically expressed in SCLC lines [14]. This highlights the potential of a higher-order regulatory network that links these key gene signatures in YAP^off^ solid cancers.

#### YAP^on^ cancers and immunotherapies

Certain YAP^on^ cancers, such as melanoma and subsets of NSCLC, respond well to immunotherapies [123,124], while others do not. Tumor mutational burden is one important factor in determining anti-tumor immunity [125], but to be targeted by CD8+ T cells, tumors must also be able to present these antigens on HLA-I [120,126]. This suggests that these ‘immune-hot’ cancers, such as melanoma, must have a combination of sufficient mutational burden along with expression of antigen processing and presentation genes—for example, the subgroup of YAP^on^ cancers with high expression of antigen processing and presentation genes [14]. In support of this notion, the majority of melanoma clusters within this subgroup [14]. This would then predict that the second subset of YAP^on^ cancers that do not express high levels of these genes and would be less immunogenic, given that they would not have the machinery to present neo-antigens even if they were present (Figure 4), although this notion requires formal testing.

Within YAP^on^ cancers, YAP also has a direct role in immune evasion, although how this relates to the two subclasses of YAP^on^ cancers (i.e., the ones with high vs. low antigen processing and presentation genes) is currently unknown. YAP can foster an immunosuppressive microenvironment through multiple mechanisms. YAP regulates the secretion of immunosuppressive cytokines and chemokines, which promote expansion and recruitment of various immunosuppressive and tumor-promoting immune cells [127–130]. YAP/TAZ also promote expression of PD-L1 on tumor cells, leading to T cell exhaustion [131–134]. Nucleotide-binding and oligomerization domain (NOD)-like receptor (NLR) family member NLRC5 is a key transcriptional regulator that promotes expression of HLA-I antigen processing and presentation genes [135]. Emerging evidence suggests that the YAP/TEAD complex represses HLA-I expression by blocking NLRC5 transcription through the recruitment of the NuRD complex [136], thereby promoting immune evasion. Further work will be required to determine whether YAP has distinct roles in controlling immune evasion in the two subsets of YAP^on^ cancers, one with high expression of antigen processing and presentation genes and one with low expression.

Additionally, MHC class II (MHC-II) molecules (known as HLA-II in humans), which are mainly expressed by professional antigen-presenting cells, such as B cells, macrophages, and dendritic cells, present exogenous peptides to CD4+ T cells [137]. While CD8+ T cells are key effectors in ICI success, CD4+ T cells are crucial for CD8+ T cell activation, memory T-cell generation, and effective ICI responses [138]. Although HLA-II is typically expressed on professional antigen-presenting cells, some tumors can also express it [139]. Tumor-specific HLA-II correlates with better prognosis, enhances immune recognition and response to ICIs, and promotes tumor rejection in preclinical models [139]. Recently, it was shown that the Hippo signaling pathway plays a role in regulating HLA-II expression in skin cancer [140]. Proteomic and transcriptomic analyses of melanoma cells revealed that Hippo pathway activation (and subsequent inactivation of YAP/TAZ) correlates with higher HLA-II expression, and knockout of these negative regulators of YAP/TAZ in cell lines resulted in up-regulation of HLA-II [140]. Furthermore, RUNX1 expression, a transcription factor that inhibits YAP/TAZ expression through its partner CBFB, is positively associated with HLA-II expression [140]. These findings suggest YAP/TAZ may play a role in suppressing HLA-II expression, thereby promoting immune evasion [140]. Taken together, these data suggest that YAP/TAZ play a crucial role in modulating HLA-I and HLA-II expression in tumors to promote tumor immune evasion and resistance to ICIs. This suggests that strategies to inhibit YAP/TAZ may also help to improve immunotherapy in certain YAP^on^ cancers, and indeed, blocking YAP/TEAD through genetic ablation or pharmacological inhibition can enhance tumor sensitivity to anti-PD-1 therapy in pre-clinical models [136]. Furthermore, in YAP^on^ cancers, combining YAP inhibitors with epigenetic therapies could further restore HLA-I expression and sensitize tumors to ICIs.

### The impact of YAP^on^/YAP^off^ class-switching and heterogeneity

Given that YAP^on^ and YAP^off^ cancers exhibit distinct therapeutic vulnerabilities, it is perhaps not surprising that tumors can convert between these different states as a mechanism of drug resistance [14]. The most well-characterized examples of this are in lung and prostate cancer, where therapy-sensitive adenocarcinomas (YAP^on^) convert to NE (YAP^off^) cancers [141–144], which has also been suggested to occur in other cancers, such as bladder [145,146] and esophageal [147]. In the prostate, this conversion has been observed in up to 25% of adenocarcinomas that develop resistance to anti-androgen therapies [143,148], and in the lung has been observed in 3–14% of adenocarcinomas that develop resistance to targeted EGFR inhibitors [141,149,150], as well as to inhibitors of ALK, ROS1, and RET kinases and immunotherapy [144]. These clinical observations match the different vulnerabilities of YAP^on^/YAP^off^ cancers, with YAP^on^ cancers exhibiting higher sensitivity to kinase inhibitors targeting these pathways [14,69,89], as well as the differences in response to immunotherapies discussed above. Thus, conversion from a YAP^on^-to-YAP^off^ state probably facilitates drug resistance in these instances. Unfortunately, the exact mechanisms that drive these conversions are poorly understood [144], and whether there is a specific role for YAP is unknown. However, given the anti-cancer role of YAP/TAZ in YAP^off^ cancers [14], it is easy to speculate that down-regulation of YAP/TAZ is likely important to permit conversion to a therapy-resistant YAP^off^ state. While this has yet to be formally tested, it could have broad clinical implications, as it may be possible to target the mechanisms that drive YAP/TAZ silencing (discussed above) to combat this route of resistance. Furthermore, it would argue that approaches to inhibit YAP/TAZ could promote YAP^on^-to-YAP^off^ cancer conversion in certain circumstances, such as combining YAP/TEAD inhibitors with EGFR inhibitors in NSCLC, which has been suggested as a potential therapeutic strategy [151]. However, it is important to note that increased YAP activity can also drive resistance to EGFR inhibitors in some instances through alternative mechanisms [152,153]. Thus, identifying biomarkers that predict which patients are at higher risk of NE/YAP^off^ conversion in this context may be an important selection criterion for these combined treatments. For example, patients with *EGFR*-mutant NSCLC are at a six-fold higher risk of NE conversion if they possess pre-existing *RB1* and *p53* mutations [150], suggesting these may not be appropriate candidates for combined therapies using EGFR and YAP/TEAD inhibitors.

In addition to converting from a YAP^on^-to-YAP^off^ state as a mechanism of drug resistance, it is possible that cancers go the opposite direction, converting from YAP^off^-to-YAP^on^. For example, it has been noted that upon autopsy, up to 25% of tumors from SCLC patients exhibit NSCLC-like histology, consistent with conversion to a YAP^on^ state [154,155]. Furthermore, in a genetically engineered mouse model of SCLC, over-expression of Myc drives temporal evolution from a YAP^off^-to-YAP^on^ state [156], and in a well-established *in vitro* model of SCLC chemotherapy resistance, the H69AR model, cells convert from a non-adherent to adherent morphology [157,158], which is accompanied by induction of YAP [20,159]. Importantly, in resistant H69AR cells, knockout of YAP impaired proliferation and tumorigenesis and partially restored sensitivity to chemotherapeutic agents, demonstrating a key role for YAP in this conversion [159]. Various other signals have also been shown to drive conversion of SCLC to an adherent, YAP^on^/NSCLC-like state, such as over-expression of mutant *RAS* or *EGFR* [160–162]. Interestingly, in these models, increased activation of AP1 transcription factors was observed [163], which is noteworthy given that AP1 co-operates with YAP/TEAD to drive oncogenesis [46,47]. Thus, interconversion between YAP^on^ and YAP^off^ states can drive tumor evolution and drug resistance in various settings, highlighting the importance of understanding the underlying mechanisms that facilitate this class-switching.

For simplicity, we have typically classified tumors as either YAP^on^ or YAP^off^; however, this state is ultimately defined at the single cell level. Thus, if a tumor primarily consists of cells in a YAP^off^ state, then the tumor will be classified as YAP^off^, and vice versa. This also means that tumors can consist of both YAP^on^ and YAP^off^ cells, which may drastically complicate treatment, given the distinct vulnerabilities of each class. In SCLC, which is primarily a YAP^off^ cancer, small patches of YAP-positive cells have been observed in some patient tumors [156], and NE foci have been noted in tumors from patients with prostate adenocarcinoma [164]. Furthermore, in mouse models of SCLC, a small percentage of non-NE, YAP-positive cells are found throughout the otherwise YAP-negative tumor [20,33,165]. Importantly, these rare YAP-positive cells are resistant to traditional chemotherapy and support the growth of the YAP^off^ bulk of the tumor [165]. Thus, YAP^on^/YAP^off^ heterogeneity has the potential to both promote tumor growth through cell-cell interactions and influence drug resistance due to the distinct vulnerabilities of each class. This suggests that combined strategies to target both states may be necessary to effectively treat these tumors. Despite this possibility, it is unknown how common this heterogeneity truly is in patients, especially across different tumor types. But given the profound clinical implications, this should be a focus of future studies.

## Conclusions

The ability to define new strategies to stratify tumors based on unique molecular signatures holds great potential to identify novel targeted therapies. However, the best way to reclassify tumors based on these molecular signatures remains to be determined. In this review, we discussed one approach, which is to functionally stratify cancers into binary YAP^on^ and YAP^off^ super-classes based on the expression and function of YAP, TAZ, and their YAP^Ad^ target genes. Importantly, these classes exhibit distinct therapeutic vulnerabilities, and cancers can interconvert between the classes as a mechanism of drug resistance, highlighting the therapeutic potential of this approach. However, it remains to be determined if cancers really can be simplified into just two groups, or if further refinement of this system is required, such as subdividing YAP^off^ cancers into liquid and solid subtypes. Furthermore, while these classes clearly exhibit distinct therapeutic vulnerabilities, we do not yet know which are the best drugs to treat each cancer class with. It is also likely that there will not be just one treatment for each class, but rather that defining a cancer as either YAP^on^ or YAP^off^ will direct you towards a subset of drugs - e.g., kinase or YAP/TEAD inhibitors for YAP^on^ cancers and BCL2, XPO1, NAMPT or epigenetic inhibitors for YAP^off^ cancers. An optimal drug may then be selected from these based on secondary features. Whether this will be additional gene signatures, such as the antigen processing and presentation signature that divides YAP^on^/YAP^off^ cancers into four subgroups, specific mutations, or other features remains to be determined, but will be an important area of future study. Leveraging this concept clinically will also require clinical trials that stratify cancers into YAP^on^/YAP^off^ classes, which thus far have not been done. HDAC inhibitors may provide the best opportunity to begin to address this concept, given that they are currently undergoing clinical trials in a wide range of cancer types [166,167].

Identifying the best way to target cancers based on YAP^on^/YAP^off^ stratification will likely be aided by better understanding the molecular basis of each class—that is, why do YAP/TAZ have opposite functions in each class and how are *YAP, TAZ,* and the YAP^Ad^ gene signature silenced in YAP^off^ cancers? For example, strategies that reactivate YAP and TAZ expression may represent a potential therapeutic approach for YAP^off^ cancers. However, we still have much to learn about the mechanisms that silence YAP/TAZ. In particular, it is unclear how conserved these mechanisms are across different YAP^off^ cancers and even samples within the same cancer type, as differences have been observed with regard to the role of various epigenetic regulators, such as LSD1, HDACs, and DNA methylation. Thus, there may be various strategies that YAP^off^ cancers use to silence these genes, and we may need to identify biomarkers for each to efficiently target them.

Mechanistically, it is still also a mystery why the YAP/TAZ-TEAD complex has completely opposite functions in solid YAP^on^ and YAP^off^ cancers. We demonstrated that this is due to the regulation of distinct transcriptional programs [14], likely because YAP/TAZ-TEAD are recruited to distinct enhancers in each cancer—cell cycle genes in YAP^on^ cancers and YAP^Ad^ genes in YAP^off^ cancers. However, why they are recruited to these distinct loci is unclear. Whether this is due to differences in chromatin organization, binding partners, or other distinct aspects between these cancer classes will require extensive genomic and proteomic studies but is critical to understanding the fundamental differences between these cancer classes. Regardless, the transcriptomic data clearly demonstrates why YAP and TAZ need to be silenced in YAP^off^ solid cancers—it is to sustain silencing of the YAP^Ad^ gene program and maintain the neural/NE lineage of these cancers, both of which are counteracted if YAP/TAZ are present. Another interesting question that arises from this is why YAP^off^ cancers do not need YAP/TAZ to drive expression of core cell cycle genes, as in YAP^on^ cancers. Clearly, other mechanisms are compensating, but what these are remains to be determined. Thus, while we have a solid foundation for understanding the molecular basis of binary YAP^on^ and YAP^off^ cancer classes, many interesting questions remain to be answered.

## Open Access

This article has been published open access under our Subscribe to Open programme, made possible through the support of our subscribing institutions, learn more here: https://portlandpress.com/pages/open_access_options_and_prices#conditional

## Abbreviations

AML: acute myeloid leukemia
AP1: activator protein 1
HLA: human leukocyte antigen
ICI: immune checkpoint inhibitor
MCC: Merkle cell carcinoma
MHC: major histocompatibility complex
NE: neuroendocrine
NEPC: NE prostate cancer
SCLC: small cell lung cancer
